# Conversion Surgery After Induction Therapy for Initially Unresectable Stage III Non-small Cell Lung Cancer: A Proof-of-Concept Trial

**DOI:** 10.1245/s10434-026-19461-z

**Published:** 2026-03-25

**Authors:** Yan Yan, Yichao Han, Zhenyi Niu, Xianfei Zhang, Chengqiang Li, Yuqin Cao, Zhengxin Yin, Lianggang Zhu, Junbiao Hang, Jiaming Che, Jian Ren, Jie Xiang, Kai Chen, Hailei Du, Dingpei Han, Xiaoqing Yang, Yajie Zhang, Xueyu Chen, Hui Jing, Shengjia Yu, Xiaofeng Wang, Zengya Guo, Xiaoyan Chen, Fangxiu Luo, Wenzhao Zhong, Runsen Jin, Hecheng Li

**Affiliations:** 1 Department of Thoracic Surgery, Ruijin Hospital, Shanghai Jiao Tong University School of Medicine, Shanghai, 200025 People’s Republic of China; 2 Institute of Respiratory Diseases, Shanghai Jiao Tong University School of Medicine, Shanghai, 200025 People’s Republic of China; 3 Department of Thoracic Surgery, West China Hospital, Sichuan University, Chengdu, 610041 People’s Republic of China; 4 Department of Pathology, Ruijin Hospital, Shanghai Jiao Tong University School of Medicine, Shanghai, 200025 People’s Republic of China; 5 Guangdong Lung Cancer Institute, Guangdong General Hospital, and Guangdong Academy of Medical Sciences, Guangzhou, 510080 People’s Republic of China

**Keywords:** Stage III NSCLC, Initially unresectable, Conversion surgery, Induction therapy, Tumor-infiltrating B cells

## Abstract

**Background:**

To prove the concept of conversion surgery after induction therapy in initially unresectable stage III non-small-cell lung cancer (NSCLC), we conducted this single-arm, open label, prospective study.

**Patients and Methods:**

In this proof-of-concept prospective study, patients with initially unresectable stage III NSCLC who had received induction therapy and evaluated radically resectable were included and underwent surgery. The primary outcome was perioperative morbidity. Bulk-RNA sequencing of baseline and post-treatment samples from patients receiving immunochemotherapy (ICT) as induction therapy was conducted to explore the transcriptomic characteristics of therapeutic response. This study was registered with ClinicalTrials.gov, NCT04945928.

**Results:**

From August 2021 to January 2024, 30 patients were enrolled. The R0 resection rate was 93.3% (28/30). Postoperative morbidity was reported in ten patients (33.3%). The rate of major pathologic response and pathological complete response were 66.7% (20/30) and 26.7% (8/30). In a median follow-up time of 17 months (10.2–21.0 months), the rate of 1-year event-free survival and overall survival were 87.5% and 96.7%, respectively. Transcriptomic analysis found a significant association of B cell immunity with MPR after ICT. A gene set derived from the enriched B cell immune-related pathways predicted better survival in patients treated with immunotherapy.

**Conclusions:**

Conversion surgery after induction therapy was safe and feasible, with encouraging 1-year survival outcomes for selected patients with initially unresectable stage III NSCLC. These foregrounded the role of radical surgery in the treatment strategy in this setting. A B cell immune-related gene set with potential predictive value was also identified within the ICT cohort.

**Clinical Trial Registry Number:**

ClinicalTrials.gov, NCT04945928.

**Supplementary Information:**

The online version contains supplementary material available at 10.1245/s10434-026-19461-z.

Lung cancer is the leading cause of cancer death worldwide.^1^ Non-small-cell lung cancer (NSCLC) is the dominant type of lung cancer.^2^ Approximately 80% of patients with NSCLC present with locally advanced or metastatic disease (stage III-IV NSCLC) that is not initially amenable to resection.^3^

Stage III NSCLC is generally categorized into resectable, potentially resectable, and unresectable. According to the latest NCCN guidelines for stage III NSCLC, resectability or potential resectability requires absence of direct invasion to surrounding vital structures and limited nodal involvement (N0, N1, or single-station N2 involvement). The European Organization for Research and Treatment of Cancer (EORTC) consensus defines unresectable stage III NSCLC as tumors with N2 bulky, N2 invasive, or N3 involvement and T4-tumors with N2 multi-station involvement.^4^ On the basis of these criteria, we further subdivide the initially unresectable stage III NSCLC into oncologically unresectable (tumors with N2 multi-station, N2 bulky, N2 invasive, or N3 involvement) and surgically unresectable (tumors directly invading vital structures precluding complete resection). For such patients, definitive concurrent chemoradiation plus durvalumab is recommended.

Conversion surgery was first reported in 1991 for limited small cell lung cancer (SCLC), which was defined as surgical resection of limited tumor after definitive medical treatment for SCLC.^5^ This concept in the treatment of initially unresectable NSCLC was also proposed, while its application was limited due to the restricted therapeutic effect. With the advances in drug therapy, an increasing number of patients with initially unresectable NSCLC were encountered with a favorable response to induction therapy.^6^ This progress converted the unresectable disease into potentially resectable status, which brought hope for radical surgery.

The reported prospective trials of conversion surgery for the potentially resectable stage IIIA-B NSCLC mostly used immunochemotherapy (ICT) as induction therapy. Results showed that this regimen could markedly enhance the rate of pathological complete response (pCR) with manageable toxic effects in stage IIIA–B NSCLC.^1,7,8^ One phase 2 study preliminarily confirmed the efficacy and safety of ICT or immunotherapy plus targeted therapy (TT) as induction therapy for these patients.^9^

Previous studies of conversion therapy for unresectable stage III NSCLC were mostly from retrospective studies.^10,11^ Only one latest published result was from a phase 2 trial.^12^ Our study therefore provides additional insights into this emerging treatment paradigm.

## Patients and Methods

### Design and Participants

This is an open-label, single-center, single-arm, prospective study (ClinicalTrials.gov registration: NCT04945928). The study protocol (Supplementary Data 1) was approved by the ethics committee of Ruijin Hospital (approval no. 2020.344). Written informed consent was obtained from all the patients prior to participation.

Patients who underwent screening were aged 18 years or older with no limit of sex, with pathologically confirmed NSCLC for tumor lesion, with N2 multi-station/bulky/invasive involvement or N3 involvement, or with direct tumor invasion to surrounding vital structures leading to incomplete resection (clinical stage T_4_N_0–3_ or T_1–3_N_2–3_ according to the 8th edition Lung Cancer Stage Classification^13^).

Screening was performed in patients who were receiving or had completed guideline-recommended systemic induction therapy [chemotherapy (ChT), TT, or ICT] in routine clinical practice, with the intent of identifying candidates for potential conversion to surgical resection. Reevaluation was performed by multidisciplinary team (MDT) for surgery viability after induction therapy. The candidate patients were enrolled only if disease was deemed surgically resectable, with tumor residual or unremissioned local lesion (transformed into R0-resectable tumor), stable or down-staged other lesions, and no distant metastasis after receiving induction therapy. Other inclusion criteria included American Society of Anesthesiologists (ASA) score of I–III, cardiopulmonary functions tolerable for of radical thoracic surgery, and normal liver and kidney functions. Patients with serious organ dysfunction leading to intolerance of the operation, with neurologic or mental illness/disorder that was hard to control, with poor compliance, who were unable to cooperate or describe the treatment response, who were unable to receive radical resection, in need of palliative or emergency operation due to lung abscess or hemoptysis, and had received neoadjuvant chemoradiotherapy were excluded.

### Treatment Procedures

At initial diagnosis and reevaluation after induction therapy, the contrast-enhanced computed tomography (CT) for chest, contrast-enhanced CT or magnetic resonance imaging (MRI) for brain, ultrasound or contrast-enhanced CT for neck and abdomen, and single-photon emission computed tomography (SPECT)-CT for bone were mandatory for clinical stage diagnosis. The positron emission tomography (PET)-CT was an optional choice. Pathological diagnosis of tumor lesion was performed by biopsy with bronchoscopy, endobronchial ultrasound (EBUS), or percutaneous puncture. Evaluation of mediastinal lymph nodes (LNs) was conducted by PET-CT or biopsy with EBUS and mediastinoscopy at baseline.

All surgeries were performed under general anesthesia with double-lumen endotracheal intubation. The specifics of each operation were at the discretion of the operating surgeon (e.g., port placement, LN dissection), as was the decision to convert to an open operation. Systematic LN dissection was routinely performed in all patients according to standard surgical principles. Surgical procedures included robot-assisted thoracic surgery (RATS), video-assisted thoracic surgery (VATS), and thoracotomy. When serious/life-threatening complications that were difficult to control occur during the procedure of RATS and VATS, the minimally invasive surgery was converted to thoracotomy.

### Outcomes

The primary endpoint was perioperative morbidity. LN count, R0 resection rate, operation time, blood loss, operative complications, postoperative hospital stay, 30-day mortality, 1-year overall survival (OS), and 1-year event-free survival (DFS) were secondary outcomes.

### Transcriptomic Analysis for Therapeutic Response

Differential expression analysis, gene set enrichment analysis (GSEA) and gene set variation analysis (GSVA) were performed in collected tumor samples. The detailed analysis procedures are in Supplementary Methods (Supplementary Data 2).

### Statistical Analysis

Because of the exploratory nature of this study, a sample size of 30 patients was determined. Categorical variables were summarized by descriptive statistics and percentage. Continuous variables following a normal distribution were presented as mean ± standard deviation (SD). In cases of noncompliance with the normal distribution, continuous variables will be presented as medians [interquartile range (IQR)].

The R package of survminer (version 0.4.9) was used to draw the survival curves. The Kaplan–Meier approach was used to calculate overall survival (OS) and event-free survival (EFS) through the R package of survival (version 3.4.0). The Cox proportional hazard regression model was used to calculate the hazard ratio (HR) and the corresponding 95% confidence interval (CI).

Bars in graphs represent mean ± standard error of mean. GraphPad Prism (version 8.0, GraphPad Software Inc., La Jolla, CA, USA), R (version 4.1.2, R Foundation for Statistical Computing, Vienna, Austria) were utilized for analyses.

## Results

### Patients

**Fig. 1:**
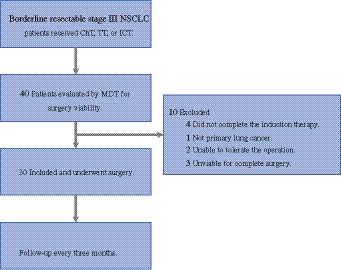
Flowchart of study enrollment

From August 2021 to January 2024, 30 eligible patients were included in Ruijin Hospital (Fig. 1); their clinical characteristics are in Table 1. The median age was 66.5 (58, 69.8) years, 25 (83.3%) patients were male, and 8 patients were current or former smokers; 9 (30%) patients had stage IIIA disease, 17 (56.7%) had stage IIIB disease, and 4 (13.3%) had stage IIIC disease. Baseline mediastinal nodal staging was PET-CT-based in all patients, with invasive confirmation in 12 (40%). All N3 cases were contralateral mediastinal nodes, pathologically confirmed in two patients. Most of the patients received ICT (22/30, 73.3%); others received ChT (4/30, 13.3%) and TT (4/30, 13.3%) as induction therapy. After induction therapy, radiographic reassessment confirmed resolution of N3 disease prior to surgery. Details of disease characteristics are summarized in Table 1.

**Table 1:** Baseline characteristics of the enrolled patients

Characteristics	(*n* = 30)
*Age*	
Median (IQR)	66.5 (58–69.8)
*Distribution*	
< 65 years	17 (56.7%)
≥ 65 years	13 (43.3%)
*Sex*	
Male	25 (83.3%)
Female	5 (16.7%)
*BMI (kg/m*^*2*^*)*	
Mean ± SD	23.36 ± 3.10
Median (IQR)	23.14 (21.05–25.13)
*Category*	
Oncologically unresectable	18 (60.0%)
Surgically unresectable	10 (33.3%)
Oncologically and surgically unresectable	2 (6.7%)
*Disease stage*	
IIIA	
T2N2M0 (multi-station N2)	4 (13.3%)
T2N2M0 (bulky/invasive N2)	1 (3.3%)
T4N0M0	4 (13.3%)
IIIB	
T3N2M0 (multi-station N2)	4 (13.3%)
T3N2M0 (bulky/invasive N2)	5 (16.7%)
T4N2M0 (multi-station N2)	2 (6.7%)
T4N2M0 (bulky/invasive N2)	2 (6.7%)
T4N2M0 (single-station, non-bulky/invasive N2)	4 (13.3%)
IIIC	
T2N3M0 (contralateral)	1 (3.3%)
T3N3M0 (contralateral)	1 (3.3%)
T4N3M0 (contralateral)	2 (6.7%)
*Clinical nodal status*	
N0	4 (13.3%)
N2	
Multi-station N2	10 (33.3%)
Bulky/invasive N2	8 (26.7%)
Single-station, non-bulky/invasive N2	4 (13.3%)
N3 (contralateral)	4 (13.3%)
*Histologic type of tumor*	
Squamous cell carcinoma	21 (70.0%)
Adenocarcinoma	5 (16.7%)
Adenosquamous cell carcinoma	1 (3.3%)
Other	3 (10.0%)
*Smoking status*	
Never smoked	22 (73.3%)
Current or former smoker	8 (26.7%)
*Type of induction therapy*	
ChT	4 (13.3%)
ICT	22 (73.3%)
TT (ALK)	3 (10.0%)
TT (EGFR)	1 (3.3%)

ChT chemotherapy, ICT immunochemotherapy, TT targeted therapy

### Surgery

Radical surgery was performed for all the included patients (Table 2), most of which was lobectomy (22/30, 73.3%); others were bilobectomy (4/30, 13.3%) and pneumonectomy (4/30, 13.3%). The R0 resection rate was 93.3% (28/30). Surgical approaches included 2 (6.7%) RATS, 14 (46.7%) VATS, and 14 (46.7%) thoracotomies. In addition, the sleeve surgery took place in six (20%) cases. Systematic ipsilateral mediastinal LN dissection was routinely performed intraoperatively, and pathological assessment was based on systematically dissected LNs at surgery. Contralateral N3 lymph nodes were not surgically removed or analyzed.

**Table 2:** Surgical outcomes

Surgical outcomes	(*n* = 30)
R0 resection	28 (93.3%)
*Surgical approach*	
Robot-assisted thoracoscopic surgery	2 (6.7%)
Video-assisted thoracoscopic surgery	14 (46.7%)
Thoracotomy	
Routine thoracotomy	5 (16.7%)
Conversion to thoracotomy	9 (30.0%)
*Surgical type*	
Lobectomy	22 (73.3%)
Bilobectomy	4 (13.3%)
Pneumonectomy	4 (13.3%)
*Reconstruction* (*n* = 6)	
Artery	2 (33.3%)
Bronchus	4 (66.7%)
*Perioperation information*	
Operation duration (min)	170 (130–220)
Estimated blood loss (ml)	200 (100–300)
Intraoperative platelet or blood transfusion	5 (16.7%)
Postoperative extubated time (days)	5 (4–6.8)
Length of hospital stay (days)	11 (9–13.8)
Length of postoperative hospital stay (days)	6 (5–8)
*Postoperative morbidity*	
Platelet or blood transfusion	2 (6.7%)
Pleural effusion	6 (20%)
Hydropneumothorax	1 (3.3%)
Atelectasis	1 (3.3%)
Pulmonary infection	2 (6.7%)
In-hospital mortality	0 (0.0%)
30-90-day mortality	1 (3.3%)

min, minute; d, day

### Safety

In total, 10 out of 30 (33.3%) patients experienced postoperative complications (Table 2), including platelet or blood transfusion (2/30, 6.7%), pleural effusion (6/30, 20%), hydropneumothorax (1/30, 3.3%), atelectasis (1/30, 3.3%), and pulmonary infection (2/30, 6.7%). No in-hospital mortality was observed, but one patient died 30-90 days after the surgery.

### Efficacy

**Fig. 2:**
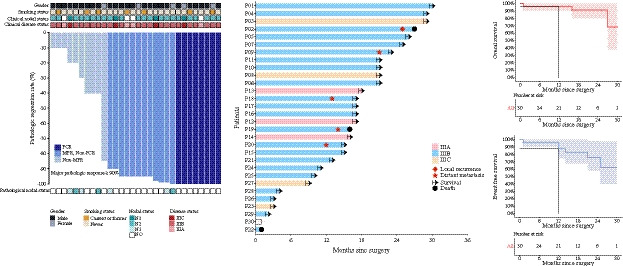
Pathological remission and survival of the 30 enrolled patients; **A** pathological remission of the 30 enrolled patients; **B** staging details, local recurrence, distant metastasis, and survival for the 30 enrolled patients; **C** event-free survival curves; **D** overall survival curves

MPR and pCR were achieved in 20 (66.7%) and 8 (26.7%) patients, respectively (Table 3). Of 26 patients with positive lymph node at baseline, 23 (88.4%) had nodal downstaging after induction therapy (Fig. 2A and Table 3). The N2 stage was further divided into N2a with single N2 station involvement and N2b with multiple N2 station involvement in the 9th edition Lung Cancer Stage Classification.^14,15^ On the basis of this lately revised N descriptors, 25 (96.2%) had nodal downstaging. The two additions were patients from N2b to N2a (Table 3).

**Table 3:** Pathological outcomes

Pathological outcomes	(n = 30)
*Pathologic response*	
pCR	8 (26.7%)
MPR	20 (66.7%)
Tumor residual > 10%	10 (33.3%)
No. of lymph nodes harvested	12.5 (9, 20)
*Downstaging of nodal status*	(n = 26)
On the basis of 8th edition Lung Cancer Stage Classification	23 (88.5%)
In patients with N3 at baseline (*n* = 23)	
N3 to N1	1 (4.3%)
N3 to N0	3 (13.0%)
In patients with N2 at baseline (*n* = 23)	
N2 to N1	3 (13.0%)
N2 to N0	16 (69.6%)
On the basis of 9th edition Lung Cancer Stage Classification	25 (96.2%)
In patients with N3 at baseline (*n* = 25)	
N3 to N1	1 (4.0%)
N3 to N0	3 (12.0%)
In patients with N2 at baseline (*n* = 25)	
N2a to N1	1 (4.0%)
N2a to N0	4 (16.0%)
N2b to N2a	2 (8.0%)
N2b to N1	2 (8.0%)
N2b to N0	12 (48%)

pCR complete pathological response, MPR major pathological response

### Survival

As of data cutoff, the median follow-up time was 17 (10.2–21.0) months (Fig. 2B). Among all the included patients, 27 (90%) are alive, and 24 (80%) are in event-free status. The rate of 1-year EFS was 87.5%, and 1-year OS was 96.7% (Fig. 2C,D).

### Predictor of Therapeutic Response and Survival

**Fig. 3:**
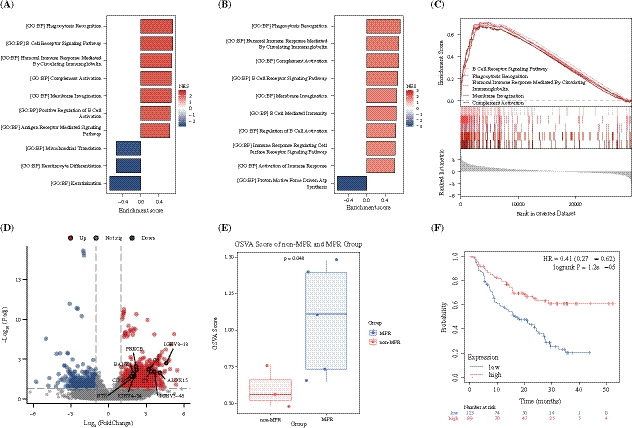
Molecular immunologic features of patients received immunochemotherapy (ICT) as induction therapy; **A** enrichment of B cell immune-related pathways in MPR compared with non-MPR post-treatment samples, **B** enrichment of B cell immune-related pathways post-treatment compared with baseline samples, **C** the top five enriched B cell immune-related pathways, **D** the top ten differentially expressed genes (DEGs) within the top five enriched B cell immune-related pathways, **E** the GSVA score of the gene set composed by the top ten DEGs in MPR and non-MPR samples, **F** the overall survival curves of patients with cancer received immunotherapy with high and low expression of the top ten DEGs

In ICT group, eight post-treatment and three baseline tumor samples, including three paired samples, were successfully collected for bulk-RNA sequencing analysis (Supplementary Data 2). The three paired samples were all from pCR group. The B cell immune-related pathways were enriched in MPR compared with non-MPR post-treatment samples (Fig. 3A) and post-treatment compared with baseline samples (Fig. 3B). A total of 66 differentially expressed genes (DEGs) were recognized within the top five enriched B cell immune-related pathways (Fig. 3C). The top ten DEGs composed a gene set (Fig. 3D), whose GSVA score was significantly elevated in MPR compared with non-MPR samples (Fig. 3E). The expression level of this gene set was also related to the OS of pan-cancer patients who received immunotherapy (Fig. 3F).^16,17^

## Discussion

Notably, this proof-of-concept study assessed the safety, feasibility, and survival outcomes of conversion therapy in patients with initially unresectable stage III NSCLC specifically. The treatment was well tolerated with manageable postoperative morbidity and satisfactory efficacy. The biomarker for responders of ICT was also revealed.

It has been controversial whether surgery could serve as a therapeutic modality for initially unresectable stage III NSCLC after downstaging with induction treatment. Durvalumab after definitive concurrent chemoradiotherapy is the standard treatment for the unresectable stage III NSCLC, which demonstrates favorable efficacy in PACIFIC study, with a 12-month progression-free survival (PFS) rate of 55.9%.^18^ Thus, the conversion surgery after induction therapy should be verified to be with a similar efficacy without drawing the unacceptable surgical-related complications.

The rates of morbidity were 13.0–40.0% in three prospective trials of neoadjuvant therapy for potentially resectable stage IIIA to IIIB NSCLC, and 13.0–16.1% in two retrospective trials of induction therapy for initially unresectable stage IIIB NSCLC.^7,8,10,11,19^ Further, 3 out of 24 patients (12.5%) who underwent surgery after induction therapy encountered surgical-related complications in a latest published exploratory study for unresectable stage II–III NSCLC.^9^ Our study demonstrated a postoperative morbidity rate of 33.3% and 30–90-day mortality rate of 3.3%, which were similar to abovementioned data, thus acceptable.

In terms of induction therapy, our regimen presented with optimistic nodal downstaging rate (26/30, 96.2%), MPR rate (20/30, 66.7%), and pCR rate (8/30, 26.7%). These short-term outcomes affirmed the efficacy of this treatment strategy.

It is widely acknowledged that the long-term prognosis of patients, including EFS and OS, is the most important criterion for evaluating the effectiveness of a cancer treatment regimen. At 12 months, the rate of EFS was 87.5%, and OS was 96.7%, which were even better than the result of PACIFIC, illustrating its potential benefit on survival.

The finding above highlighted and advanced the position of radical surgery in the treatment strategy of initially unresectable stage III NSCLC.

In addition, regimens of induction therapy in our study were ICT, ChT, and TT. Among them, ICT was the most common therapy in our study and also in other trials, exhibiting a favorable rate of MPR (16/22, 72.7%) and pCR (6/22, 27.3%). The MPR/pCR rate was 100% (4/4)/75% (3/4) in TT group and 33.3% (1/4)/0.0% (0/4) in ChT group. Unfortunately, further comparison was unavailable due to the great difference of sample size in the three groups, which could be the topic for future discoveries.

Regarding potential biomarkers for responders of ICT, we identified a B cell immune-related gene set, which may serve as a promising predictor for the MPR and OS. It is commonly believed that cytotoxic T cells are the directors of antitumor immunity in tumor microenvironment (TME), which made them the core of most tumor immunology research. Notably, the tumor-infiltrating B lymphocytes (TIBs) are also essential members of tumor-infiltrating lymphocytes (TILs), which carry strong prognostic significance.^20^ B cell regulates tumor-driven antibody-mediated immune responses through mechanisms of complement dependent cytotoxicity (CDC), antibody-dependent cell mediated phagocytosis (ADCP), and antibody-dependent cell mediated cytotoxicity (ADCC). Previous results have shown that B cell infiltration in NSCLC was significantly higher than that in nontumoral tissue.^21^ Follicular B cells, plasma cells, and germinal center (GC) B cells were the main subsets of TIBs in NSCLC with their own gene signatures, of which the plasma cells were identified to be predictor of OS in patients treated with atezolizumab, but not chemotherapy.^22^ The components of our B cell immune-related gene set were all included in the three B cell subsets signatures identified in the aforementioned analysis. We believe this is the first study to verify enrichment of B cell immune-related pathways in MPR post-treatment NSCLC samples, which proves the close relationship between B cell immunity and therapeutic response. Additionally, the gene set we produced has strong potential to be the predictor of survival for patients who received immunotherapy.

This study has several limitations. Firstly, it adopts a single-arm study design, lacking data from a control group undergoing durvalumab after definitive concurrent chemoradiotherapy. Secondly, the participants received three different patterns of induction therapy, which increased the heterogeneity. Third, invasive mediastinal staging was performed in only a subset of patients at baseline, whereas post-induction nodal status was confirmed by pathological assessment of systematically dissected lymph nodes at surgery. Therefore, nodal downstaging, defined by comparing baseline nodal staging with postoperative pathological nodal status, should be interpreted with caution, particularly in patients whose baseline nodal status was determined radiographically. Another limitation is that this study was entirely based on the single-center population, thus limiting the generalizability of the results. In addition, the relevance between the B cell immune-related gene set and B cell antitumor immunity in TME should be verified in future research. These gaps lay the groundwork for further investigation in future research.

## Conclusions

Conversion surgery after induction therapy was safe and feasible for selected patients with initially unresectable stage III NSCLC with acceptable surgical-related morbidity, favorable MPR/pCR rate, and encouraging 1-year DFS/OS outcomes. These findings foregrounded the role of radical surgery in the multidisciplinary treatment strategy for initially unresectable stage III diseases. Within the ICT cohort, enrichment of B cell-related immune pathways in MPR samples identified a B cell immune gene set associated with survival in an independent immunotherapy-treated cohort, highlighting its potential relevance to immunotherapy response.

## Supplementary Information

Below is the link to the electronic supplementary material.

Supplementary file1 (DOCX 976 KB)

Supplementary file2 (DOCX 28 KB)

## Data Availability

The data underlying this article will be shared on reasonable request to the corresponding author.
