# Supplementary material for: Conversion Surgery After Induction Therapy for Initially Unresectable Stage III Non-small Cell Lung Cancer: A Proof-of-Concept Trial
Source: Ann Surg Oncol. 2026 Mar 25;33(7):6242–51. doi: 10.1245/s10434-026-19461-z (PMC13242421; doi:10.1245/s10434-026-19461-z)
Supplement: Supplementary file 1 — Supplementary file1 (DOCX 976 KB) [file 10434_2026_19461_MOESM1_ESM.docx]

# TITLE PAGE

**Safety and Feasibility of Surgery After Conversion Therapy for**

**Locally Advanced and Advanced NSCLC**

**Principal Investigator:** Hecheng Li, M.D., Ph.D

Professor of Thoracic Surgery

Department Chair of Thoracic Surgery

Ruijin Hospital

Shanghai Jiao Tong University School of Medicine

Address: 197 Ruijin Er Road, Shanghai (200025), China

Tel: 86-21-64370045*664566(O)

E-mail: [lihecheng2000@hotmail.com](mailto:lihecheng2000@hotmail.com)

Edition number: V1.0

Approval time: June 22, 2021

TABLE OF CONTENTS

[TITLE PAGE 1](#_Toc149583016)

[LIST OF ABBREVIATIONS AND DEFINITIONS OF TERMS 4](#_Toc149583017)

[1. PROTOCOL SUMMARY 6](#_Toc149583018)

[1.1 Synopsis 6](#_Toc149583019)

[1.2 Schema 8](#_Toc149583020)

[2. INTRODUCTION 9](#_Toc149583021)

[2.1 Background and rationale for conducting this study 9](#_Toc149583022)

[2.2 Rationale for study design, study population and control group 9](#_Toc149583023)

[2.3 Benefit/risk and ethical assessment 10](#_Toc149583024)

[3. STUDY OBJECTIVES, ENDPOINTS AND DEFINITIONS 11](#_Toc149583025)

[4. PATIENT ELIGIBILITY, ENROLLMENT, RANDOMIZATION, AND WITHDRAWAL 12](#_Toc149583026)

[4.1 Patient eligibility 12](#_Toc149583027)

[4.2 Patient enrollment and randomization 12](#_Toc149583028)

[4.3 Withdrawal of study 14](#_Toc149583029)

[5. TREATMENT METHODS 14](#_Toc149583030)

[5.1 Preoperative investigation and preparation 14](#_Toc149583031)

[5.2 Surgery 14](#_Toc149583032)

[5.3 Postoperative management 15](#_Toc149583033)

[5.4 Postoperative adjuvant therapy 16](#_Toc149583034)

[6. ASSESSMENT AND DATA COLLECTION 16](#_Toc149583035)

[6.1 Preoperative assessments and data collection 16](#_Toc149583036)

[6.2 Operative assessments and data collection 16](#_Toc149583037)

[6.3 Pathology assessment 17](#_Toc149583038)

[6.4 Postoperative assessment and data collection 17](#_Toc149583039)

[6.5 Follow-up assessment and data collection 17](#_Toc149583040)

[6.6 Assessment of health-related quality of life and postoperative pain 17](#_Toc149583041)

[6.7 Complications and safety reporting 17](#_Toc149583042)

[7. STATISTICAL CONSIDERATIONS 20](#_Toc149583043)

[7.1 Statistical hypotheses 20](#_Toc149583044)

[7.2 Sample size estimation 20](#_Toc149583045)

[7.3 Statistical analysis principles 20](#_Toc149583046)

[7.4 Missing data strategy 21](#_Toc149583047)

[7.5 Statistical analysis set 21](#_Toc149583048)

[7.6 Interim analysis 21](#_Toc149583049)

[8. ETHICAL CONSIDERATIONS 21](#_Toc149583050)

[8.1 Responsibilities of investigators 21](#_Toc149583051)

[8.2 Compensation 21](#_Toc149583052)

[9. REFERENCES 21](#_Toc149583053)

[10. APPENDICES 23](#_Toc149583054)

[Appendix 1 The eighth edition of the TNM classification for lung cancer 24](#_Toc149583055)

[Appendix 2 The American Society of Anesthesiologists (ASA) Physical Status Classification 26](#_Toc149583056)

[Appendix 3 English model of informed consent form 27](#_Toc149583057)

[Appendix 4 The Clavien-Dindo classification of surgical complication 31](#_Toc149583058)

[Appendix 5 English model of serious complication report form 32](#_Toc149583059)

# LIST OF ABBREVIATIONS AND DEFINITIONS OF TERMS

| **Abbreviation/Term** | **Definition/Explanation** |
| --- | --- |
| ALT | alanine aminotransferase |
| AP | anteroposterior |
| ASA | American Society of Anesthesiologists |
| AST | aspartate aminotransferase |
| CA19-9 | carbohydrate antigen 19-9 |
| CA72-4 | carbohydrate antigen 72-4 |
| CEA | carcinoembryonic antigen |
| CI | confidence interval |
| CRFs | case report forms |
| CRP | C-reactive protein |
| CT | computed tomography |
| DFS | disease-free survival |
| EBUS | endobronchial ultrasound |
| ECG | echocardiography |
| EORTC QLQ-C30 | the European Organization for Research and Treatment of Cancer Quality of Life Questionnaire |
| EORTC QLQ-LC13 | the European Organization for Research and Treatment of Cancer Quality of Life Questionnaire in Lung Cancer |
| EQ-5D | the European Quality of Life 5 Dimensions |
| ERAS | enhanced recovery after surgery |
| FDG | fluorodeoxyglucose |
| FEV1 | forced expiratory volume in one second |
| FEV1/FVC | forced expiratory volume in one second/forced vital capacity |
| FVC | forced vital capacity |
| GCP | good clinical practice |
| Hb | hemoglobin |
| HRQoL | health-related quality of life |
| ICF | informed consent form |
| ICS | intercostal space |
| IQR | interquartile range |
| IRB | institutional review board |
| ITT | intent-to-treat |
| LN | lymph node |
| LYM | lymphocyte count |
| MVV | maximal voluntary ventilation |
| NRS | numeric rating scale |
| NSCLC | non-small cell lung cancer |
| NSE | neuron-specific enolase |
| OS | overall survival |
| PET/CT | positron emission tomography/computed tomography |
| PLT | platelet count |
| PP | per-protocol |
| RATS | robotic-assisted thoracic surgery |
| SD | standard deviation |
| VAS | visual analog score |
| VATS | video-assisted thoracic surgery |
| WBC | white blood cell count |
| WHO | World Health Organization |

# 1. PROTOCOL SUMMARY

## 1.1 Synopsis

**Protocol Title**

Safety and Feasibility of Surgery After Conversion Therapy for Locally Advanced and Advanced NSCLC.

**Objective**

The aim of this study is to evaluate the safety and feasibility of conversion surgery after induction therapy for locally advanced and advanced non-small cell lung cancer (NSCLC).

**Endpoints**

| Endpoints | Definition |
| --- | --- |
| Primary endpoints |  |
| Perioperative morbidity | The percentage of patients who have perioperative complications, mainly include: pneumonia, arrhythmia, incision infection, vocal cord paralysis, trachea cannula |
| Main secondary endpoints |  |
| Lymph nodes count | Overall lymph node counts, number of stations dissected, and number of lymph nodes in each lymph node station |
| R0 rate | The percentage of patients who achieve R0 resection. R0 is a curative resection with negative resection margins. |
| Operation time | The time from skin incision to skin closure. |
| Blood loss | The volume of blood lost in the operation. |
| Operative complications | The rate of conversion to open surgery in the operation |
| Postoperative hospital stay | Postoperative hospital stay measures the length of time elapsed between a patient’s date of surgery and discharge. |
| 30-day mortality | Death within 30 days of the surgical procedure, including patients who die after discharge from hospital. |
| 1-year overall survival (OS) | The percentage of patients who are still alive 3 years after intervention. |
| 3-year overall survival (OS) | The percentage of patients who are still alive 3 years after intervention. |
| 1-year disease-free survival (DFS) | The percentage of patients who are still alive without any evidence of disease 1 years after randomization. |
| 3-year disease-free survival (DFS) | The percentage of patients who are still alive without any evidence of disease 3 years after randomization. |

**Overall Design**

This is a single-arm, prospective study (ClinicalTrials.gov number, NCT04945928) to evaluate the safety and feasibility of conversion surgery after induction therapy for locally advanced and advanced non-small cell lung cancer.

**Design Outline**

***Patients***

| Inclusion criteria | Exclusion criteria |
| --- | --- |
| 1. Volunteer to participate in the study with good compliance. Able to complete the observation and follow-up and have signed the informed consent; 2. More than 18 years old with no limit of sex; 3. Pathologically confirmed (tumor lesion) T4N0-3 or T1-3N2-3 NSCLC with residual tumor after first-line treatment (first-line chemotherapy, targeted therapy, immunotherapy, chemotherapy combined with immunotherapy, etc.) evaluated operable for radical resection; pathologically confirmed stage IVA NSCLC evaluated operable after first-line treatment (first-line chemotherapy, targeted therapy, immunotherapy, chemotherapy combined with immunotherapy, etc.); 4. ASA score: I-III; 5. Cardiopulmonary functions meet the requirements of radical operation for lung cancer with normal liver and kidney functions. | 1. Serious heart, lung, liver and kidney dysfunction and unable to tolerate the operation; 2. Neurologic, mental illness or mental disorder which is hard to control, poor compliance, unable to cooperate or describe the treatment response; 3. Unable to receive radical resection; 4. Need of palliative or emergency operation due to lung abscess or hemoptysis; 5. Having received neoadjuvant chemoradiotherapy. |

***Sample size***

Regarding the exploratory nature of this study, a sample size of 30 patients was determined.

***Participant Group/Arm***

Participants with locally advanced or advanced NSCLC who received first-line treatment have been evaluated as resectable after multidisciplinary discussion involving the department of thoracic surgery, respiratory medicine, radiology, and oncology.

***Intervention/Treatment***

Participants having been evaluated as operable after receiving first-line treatment (first-line chemotherapy, targeted therapy, immunotherapy, chemotherapy combined with immunotherapy, etc.) will receive conversion surgery.

***Statistical Analysis***

Continuous variables which is normally distributed will be presented as mean ± standard deviation, and Student’s t-test will be used for comparison in subgroup analysis. In cases of non-compliance, continuous variables will be presented as median (interquartile range), and compared by Wilcoxon rank-sum test between subgroups. Categorical variables were presented as frequency with percentage. The test level between the subgroups will be set at α=0.05 (bilateral), and differences with P <0.05 will be considered to be statistically significant.

## 1.2 Schema

# 2. INTRODUCTION

Lung cancer is the leading cause of cancer death worldwide^1^. An estimated 2.2 million new lung cancer cases occurred in 2020, accounting for about 11.4% of total cancer diagnoses. NSCLC is the dominant type of lung cancer^2^. Approximately 80% of patients with NSCLC present with either metastatic or locally advanced disease that is not initially amenable to resection^3^.

Treatments for locally advanced NSCLC, stage III NSCLC, is controversial. Surgical resection is primarily recommended for T1-3 diseases with single station and non-bulky N2, while other stage III NSCLC are determined initially unresectable. Previous studies have shown that conversion surgery after induction therapy could bring potential benefits for these patients^4-8^. However, it requires strict operation indications and patient screening. Furthermore, this concept of treatment still needs evidence from prospective studies to prove its safety and feasibility.

This single-arm, prospective study evaluated the safety and feasibility of conversion surgery after induction therapy for initially unresectable locally advanced and advanced NSCLC patients.

## 2.1 Background and rationale for conducting this study

This study is designed to evaluate the safety and feasibility of surgery after conversion therapy for locally advanced and advanced non-small cell lung cancer.

## 2.2 Rationale for study design and study population

This is a single-center, single-arm, and prospective study evaluated the safety and feasibility of surgery after conversion therapy for initially unresectable locally advanced and advanced NSCLC patients.

Locally advanced and advanced NSCLC are stage III-IV diseases. According to the latest NCCN guidelines for NSCLC, selected T_1-3_N_2_ NSCLC patients with single station and non-bulky N_2_ can receive systemic therapy first, and if there’s no apparent progression, they may be considered to receive surgery. The T_3-4_N_1-0_ NSCLC patients may also be considered to undergo surgery, while the resectability should be well evaluated. Others are initially unresectable, and multidisciplinary treatments such as chemoradiation followed by Durvalumab and targeted therapy are recommended.

Conversion surgery is a kind of salvage surgery in a broad sense. Salvage surgery for lung cancer was first reported in 1991 for limited small cell lung cancer, which was defined as surgical resection of limited tumor after definitive medical treatment for small cell lung cancer (SCLC)^9^. Thanks to the advances in drug therapy and radiotherapy for NSCLC, conversion surgery for initially unresectable NSCLC was proposed and evaluated rapidly. This surgery is for NSCLC converted from initially unresectable to potentially resectable status because of a favorable response to induction therapy^10^.

The latest results from prospective trials of conversion surgery were all for the stage IIIA-B NSCLC. The immunotherapy plus chemotherapy was used as induction therapy. Results showed that this regimen could markedly enhance the rate of pathological complete response (pCR) with manageable toxic effects in stage IIIA-B NSCLC^4-6^.

Current reported results of conversion surgery for later stage locally advanced and advanced NSCLC were all from retrospective studies^7,8^. There continues to be a lack of evidence from prospective trials to verify its safety and feasibility. Therefore, we started this single-arm, prospective study to evaluate the safety and feasibility of conversion surgery after induction therapy for initially unresectable locally advanced and advanced NSCLC patients.

**2.2.1 Rationale for study population**

This study is a single-arm clinical trial. The aim of this study is to evaluate the safety and feasibility of salvage surgery after induction therapy for locally advanced and advanced non-small cell lung cancer. Due to the lack of previous studies for reference, the sample size is not calculated based on hypothesis testing. Regarding the exploratory nature of this study, a sample size of 30 patients was determined.

**2.2.2 Rationale for study patient**

Patients with initially unresectable NSCLC having been evaluated as operable after receiving first-line treatment are eligible for the study.

Initially unresectable clinical stage IIIA-B NSCLC is defined as T_1-3_ with multi station or bulky N_2_ and T_3-4_ N_1-0_ with invasion. All the clinical stage IIIC and IV NSCLC are initially unresectable. The Appendix 1 displays the eighth edition of the TNM classification for lung cancer.

The first-line treatments include chemotherapy, chemotherapy plus immunotherapy, and targeted therapy.

Previous studies have shown that conversion surgery after induction therapy could bring potential benefits for these patients^4-8^. Therefore, we included these patients to receive conversion surgery in our study.

## 2.3 Benefit/risk and ethical assessment

**2.3.1 Benefit assessment**

Conversion surgery after induction therapy has been shown to be a safe and feasible treatment for patients with initially unresectable NSCLC in retrospective studies. Additionally, this therapy may result in better pCR, disease-free survival (DFS), and overall survival (OS).

**2.3.2 Risk assessment**

There are disadvantages of conversion surgery after induction therapy, which could form the risks and disadvantages associated with participation in this study. The main risks for the patients are from surgery-related complications. The risks of these complications will be minimized by dedicated surgery teams. The qualifications of the responsible surgeons that participate in this study are described in section 5.2.1.

**2.3.3 Overall benefit risk conclusion**

Taking into account the measures taken to minimize risks to participants in this study, the potential risks identified in association with conversion surgery after induction therapy are justified by the anticipated benefits that may be afforded to participants with initially unresectable NSCLC.

**2.3.4 Ethical assessment**

This study will be carried out upon approval of the Institutional Review Board (IRB) in Ruijin Hospital and in compliance with the Helsinki Declaration and Good Clinical Practice (GCP) guidelines.

In keeping with the related regulations and guidelines, subjects can participate in the study only after having signed the ICF in full awareness of the objectives of the study and the risks involved and each subject has the right to withdraw from the study at any time.

We will ensure confidentiality of all patient- and study-related information and explain to the subjects the details of compensation.

# 3. STUDY OBJECTIVES, ENDPOINTS AND DEFINITIONS

**3.1 Objectives**

The aim of this study is to evaluate the safety and feasibility of conversion surgery after induction therapy for locally advanced and advanced non-small cell lung cancer.

**3.2 Endpoints and definitions**

| Endpoints | Definition |
| --- | --- |
| Primary endpoints |  |
| Perioperative morbidity | The percentage of patients who have perioperative complications, mainly include: pneumonia, arrhythmia, incision infection, vocal cord paralysis, trachea cannula |
| Main secondary endpoints |  |
| Lymph nodes count | Overall lymph node counts, number of stations dissected, and number of lymph nodes in each lymph node station |
| R0 rate | The percentage of patients who achieve R0 resection. R0 is a curative resection with negative resection margins. |
| Operation time | The time from skin incision to skin closure. |
| Blood loss | The volume of blood lost in the operation. |
| Operative complications | The rate of conversion to open surgery in the operation |
| Postoperative hospital stay | Postoperative hospital stay measures the length of time elapsed between a patient’s date of surgery and discharge. |
| 30-day mortality | Death within 30 days of the surgical procedure, including patients who die after discharge from hospital. |
| 1-year overall survival (OS) | The percentage of patients who are still alive 3 years after intervention. |
| 3-year overall survival (OS) | The percentage of patients who are still alive 3 years after intervention. |
| 1-year disease-free survival (DFS) | The percentage of patients who are still alive without any evidence of disease 1 years after randomization. |
| 3-year disease-free survival (DFS) | The percentage of patients who are still alive without any evidence of disease 3 years after randomization. |

# 4. PATIENT ELIGIBILITY, ENROLLMENT, RANDOMIZATION, AND WITHDRAWAL

## 4.1 Patient eligibility

Each subject should meet all of the inclusion criteria and none of the exclusion criteria for this study. Under no circumstances can there be exceptions to this rule.

**4.1.1 Inclusion criteria**

For inclusion in the study, subjects should fulfil the following criteria:

1. Volunteer to participate in the study with good compliance. Able to complete the observation and follow-up and have signed the informed consent;
2. More than 18 years old with no limit of sex;
3. Pathologically confirmed stage T_4_N_0-3_ or T_1-3_N_2-3_ NSCLC with residual tumor after first-line treatment (first-line chemotherapy, targeted therapy, immunotherapy, chemotherapy combined with immunotherapy, etc.) evaluated operable for radical resection; pathologically confirmed stage IVA NSCLC evaluated operable after first-line treatment (first-line chemotherapy, targeted therapy, immunotherapy, chemotherapy combined with immunotherapy, etc.);
4. ASA score: I-III;
5. Cardiopulmonary functions meet the requirements of radical operation for lung cancer with normal liver and kidney functions.

**4.1.2 Exclusion criteria**

Patients should not enter the study if any of the following preoperative exclusion criteria are fulfilled:

1. Serious heart, lung, liver and kidney dysfunction and unable to tolerate the operation;
2. Neurologic, mental illness or mental disorder which is hard to control, poor compliance, unable to cooperate or describe the treatment response;
3. Unable to receive radical resection;
4. Need of palliative or emergency operation due to lung abscess or hemoptysis;
5. Having received neoadjuvant chemoradiotherapy.

## 4.2 Patient enrollment

**4.2.1 Patient selection**

Clinical examination data of patients conducted from hospital admission to enrollment into this study (time period is usually 1 week) will be considered baseline data, and must include:

1. Systemic status: height, weight, comorbidities;
2. Peripheral venous blood: white blood cell count (WBC), hemoglobin (Hb), platelet count (PLT), lymphocyte count (LYM);
3. Blood biochemistry: albumin, prealbumin, total bilirubin, AST, ALT, creatinine, fasting glucose, CRP;
4. Serum tumor markers: CEA, CA19-9, CA72-4, NSE;
5. Contrast-enhanced chest computed tomography (CT) (slice thickness of 2.5 mm or less; in case of allergy to the contrast agent, non-contrast CT is allowed);
6. Chest X-ray (AP and lateral views): cardiopulmonary conditions;
7. Resting 12-lead ECG;
8. Respiratory function tests: FEV1, FVC, FEV1/FVC, MVV.

For patients with other comorbidities (e.g., diabetes, coronary heart disease, hypertension), corresponding examinations will be performed to exclude surgical contraindications.

For solid pulmonary nodules on normal chest CT, an FDG PET/CT scan is warranted to screen out aggressive, advanced-stage tumors. If PET/CT scan is positive in the mediastinum, lymph node status needs pathologic confirmation. Methods for pathologic confirmation of lymph node status include mediastinoscopy and endobronchial ultrasonography (EBUS).

After the above-mentioned preoperative examination, a surgery team led by Hecheng Li will discuss the possibility of surgery and how it will be done.

**4.2.2 Informed consent**

Patients will be approached for possible recruitment following clinical evaluation, provided they fulfil the patient eligibility criteria (see section 4.1). Patients who fail to meet the patient eligibility criteria should not, under any circumstances, be enrolled in this study. There can be no exceptions to this rule. Patients will be provided with verbal and written details. A verbal explanation of the trial along with the ICF will be provided by a medically qualified member of the surgery team for the patient to consider (Appendix 3). The rationale, design and personal implications of the trial will be provided and explained by the study member.

Following information provision, patients should be given the opportunity to discuss the trial with their family and healthcare professionals before they are asked whether they are willing to take part in the trial. Patients will be given as much time as possible to consider their participation in the trial. The right of the patient to refuse consent without giving reasons will be respected.

Assenting patients will then be formally assessed for eligibility and invited to provide informed written consent for their participation in the study. ICF may only be obtained by the principal investigator or another clinically qualified member of the trial team who has received GCP training and is approved by the principal investigator to take informed consent as documented in the trial log.

The patient consent form with all original signatures must be retained in the trial log. A copy of the signed ICF should be given to the patient, and a record of the consent process, detailing the date of consent and witnesses, should also be kept in the patient’s notes (this may include a copy of the ICF as per local practice).

Patients will remain free to withdraw from the trial at any time by revoking consent without giving reasons and without prejudicing any further treatment.

## 4.3 Withdrawal of study

At any time, patients are free to withdraw from the study without prejudice to further treatment. For patient who withdraws between inclusion and surgery, the patient will be excluded from the study and no further data of the patients will be recorded. For patients withdrawing after surgery, perioperative outcomes will still be recorded. They will be asked if they are willing to continue with survival follow-up (which can be conducted by telephone). If the patients wish to withdraw from the study entirely, including further follow-up, this should be clearly documented in the patient notes.

# 5. TREATMENT METHODS

## 5.1 Preoperative investigation and preparation

Preoperative investigation and preparation will be accorded with the routine clinical work in the department of thoracic surgery, Ruijin Hospital affiliated Shanghai Jiao Tong University School of Medicine (see section 4.2.1).

## 5.2 Surgery

All surgeries will be performed under general anesthesia with double-lumen endotracheal intubation. The specifics of each operation will be at the discretion of the operating surgeon (e.g., port placement, lymph node dissection), as will the decision to convert to an open operation. There are, however, basic standard of surgical procedures, which are described in the following sections.

**5.2.1 Robot-assisted thoracic surgery (RATS)**

RATS is performed using a da Vinci S/Si surgical robot (Intuitive Surgical, Inc, Santa Clara, CA). Five ports are placed in the following positions: a 12-mm camera port is placed in the eighth ICS at the mid-axillary line; three 8-mm working ports are placed separately in the fifth ICS at the anterior axillary line (#1 arm), the eighth ICS at the posterior axillary line (#2 arm), and the eighth ICS at 2 cm lateral to the spine (#3 arm). Finally, the auxiliary port is placed in the eighth ICS between the camera port and the anterior port.

**5.2.2 Video-assisted thoracic surgery (****VATS)**

VATS is performed through a 4-cm incision, which is placed in the fifth ICS at the anterior axillary line and covered with a protective sleeve. Uniportal VATS is preferred over multiportal VATS. When necessary, an additional auxiliary port will be placed in the sixth or eighth ICS at the mid-axillary line. All surgical instruments are inserted through the incision without spreading the ribs.

**5.2.3 Thoracotomy**

Thoracotomy is performed through muscle sparing (MS) incision, which is placed in the fifth ICS at the anterior axillary line.

**5.2.4 Regulations on conversion to** **thoracotomy**

When intra-thoracic hemorrhage, organ damage and other serious/life-threatening complications which are difficult to control occur during the procedure of RATS and VATS, it is necessary to actively convert to thoracotomy. The surgeon in charge can decide to convert to thoracotomy for other technical or equipment reasons. The reasons for all cases of conversion to thoracotomy must be clearly recorded in the case report forms (CRFs). The incision length for the conversion to thoracotomy is not regulated in this study.

**5.2.5 Lymph node dissection**

Appropriate N1 and N2 lymph node stations should be dissected for each patient. Mediastinal nodal staging must be accomplished by dissection of a minimum of three N2 stations for all patients.

## 5.3 Postoperative management

Enhanced recovery after surgery (ERAS) is routinely implemented, including smoking cessation, breathing training, analgesia, early postoperative activities, early postoperative extubation, etc.

**5.3.1 Preventive use of analgesics**

Patient-controlled analgesia and oral nonsteroidal anti-inflammatory drugs are given routinely for postoperative analgesia. Intravenous nonsteroidal anti-inflammatory drugs, meperidine, and tramadol are used as extra-analgetic according to specific patient conditions.

**5.3.2 Preventive use of antibiotics**

Postoperative prophylactic intravenous antibiotics is routinely used, which are the second generation cephalosporins. For patients who are allergic to cephalosporins, clindamycin will be used instead. The preventive use of antibiotics usually lasts for 24 hours, and hardly exceeds 48 hours. The specific use of antibiotics will be based on doctor’s experience and routine practices.

**5.3.3 Fluid replacement**

Postoperative fluid infusion (including glucose, insulin, electrolytes, vitamins, etc.) will be performed based on doctor’s experience and routine clinical practices, and is not specified in this study. After oral feeding, it is allowable to stop or gradually reduce fluid infusion/nutritional support.

**5.3.****4 Discharge**

The nursing and rehabilitation therapists ensure that patients are mobilized on the day of surgery or as soon as possible. With the help of the rehabilitation therapists, the patients are encouraged to do the chest expansion exercises, segmental breathing, and respiratory muscle training. In the absence of postoperative complications, abnormality on postoperative chest X-rays and fever, the patient can be discharged, which should be recorded in the CRFs. On discharge, patients are asked to contact the thoracic ward if concerns arise.

## 5.4 Postoperative adjuvant therapy

The principle of adjuvant therapy is in accordance with the national comprehensive cancer network guideline for NSCLC^11^. Postoperative adjuvant therapy is recommended for patients with high-risk pathological stage IB, and pathological stage IIA or later stages. High-risk factors may include poorly differentiated tumors (including lung neuroendocrine tumors [excluding well-differentiated neuroendocrine tumors]), vascular invasion, visceral pleural involvement. These factors independently may not be an indication and may be considered when determining treatment with adjuvant chemotherapy. For patients who have indications for adjuvant therapy, adjuvant therapy is recommended but not mandatory, which is at discretion of the patients. As the guideline is keeping updating, the indication and regimen of adjuvant therapy may change as well. In general, we will follow the latest guideline, although there may be some differences due to the unavailability of certain drugs.

# 6. ASSESSMENT AND DATA COLLECTION

## 6.1 Preoperative assessments and data collection

Preoperative investigation and preparation will be accorded with the routine clinical work in the department of thoracic surgery, Ruijin Hospital affiliated Shanghai Jiao Tong University School of Medicine (see section 4.2.1).

Data collected on the randomization, eligibility and preoperative CRFs will include:

1. Personal details and demographics including height, weight, and gender;
2. Preoperative investigations performed;
3. Clinical TNM staging (the eighth edition of the TNM classification for lung cancer^12^, Appendix 1);
4. Confirmation of eligibility;
5. Confirmation of written informed consent;
6. Date of randomization;
7. Known concomitant diseases and co-morbidities;
8. Smoking status (smoking index (number of cigarettes smoked per day multiplied by years of tobacco use) is used to evaluate the smoking status of the patient).

## 6.2 Operative assessments and data collection

The part of operative information in the CRFs will be completed. This collects data relating to the operation including:

1. Surgeon;
2. ASA status;
3. Thoracoscopic technique (robotic-assisted or video-assisted);
4. Whether there is an intraoperative change of surgical plan (inappropriateness for minimally invasive lobectomy based on intraoperative exploration, such as sublobar resection for a small lesion or extensive lung resection for a massive tumor);
5. Duration of operation;
6. Whether outcome of operation curative, palliative or unresectable;
7. Whether conversion to open surgery occurred, and reason;
8. Any intraoperative complications.

## 6.3 Pathology assessment

The pathology assessment is accorded with the 2015 World Health Organization (WHO) Classification of Tumors of the Lung, Pleura, Thymus, and Heart^13^. The part of pathology information in the CRFs will be completed. This collects data relating to the pathology including:

1. Gross description including sample size and tumor size;
2. Histology including type and differentiation;
3. Metastatic spread including lymph nodes (number retrieved and number involved), lymphatic or vascular invasion, neural invasion, pleural invasion;
4. Co-existent conditions (e.g., granuloma necrosis);
5. Pathological TNM stage (the eighth edition of the TNM classification for lung cancer^12^, Appendix 1).

## 6.4 Postoperative assessment and data collection

Postoperative care has been described before (see section 5.3). A postoperative clinical assessment must be carried out for all patients and the data collected will include:

1. Duration of postoperative hospital stay (date fit for discharge, actual discharge date, reason for any delay);
2. Postoperative complications and severity;
3. Details of any further surgery or invasive operation required and reason.

## 6.5 Follow-up assessment and data collection

Follow-up data will be collected every 6 months after surgery until patient death or the completion of the study.

Data collected will include:

1. Patient status (alive or dead) and details of death, including:

- Date of death
- Reason of death (NSCLC related or other)

1. Details of any adjuvant therapy;
2. Details of any local or distant recurrence, including:

- Date of recurrence
- Site of recurrence
- Method of diagnosis
- Treatment for recurrence

## 6.6 Assessment of health-related quality of life and postoperative pain

Patients will be asked to complete the generic HRQoL and pain questionnaires.

For the assessment of HRQoL, three HRQoL instruments are used: the European Organization for Research and Treatment of Cancer Quality of Life Questionnaire (EORTC QLQ-C30) version 3.0^14^, the EORTC Quality of Life Questionnaire in Lung Cancer (EORTC QLQ-LC13),^15^ and the European Quality of Life 5 Dimensions (EQ-5D) questionnaire.^16^ This will be done before surgery and at weeks 4, 24, and 48 postoperatively.

For pain evaluation, an 11-point visual analog score (VAS) (on postoperative day 1) and numeric rating scale (NRS) (used during follow-up) are used, with a score of 0 representing “no pain” and a score of 10 signifying the “worst pain imaginable”. The pain assessment is done at week 4, 24, and 48 postoperatively.

## 6.7 Complications and safety reporting

For the purpose of this trial, the safety reporting terms adverse events and serious adverse events have been translated into complications.

**6.7.1 General definitions**

A complication is defined as an untoward medical event in a patient, which has a causal relationship to the trial. The trial includes the surgical intervention and procedures directly related to the surgery (e.g., anesthetic) and any trial specific interventions (e.g., the consent process and completion of questionnaires).

The grade of complications is evaluated using the Clavien-Dindo classification (Appendix 4),^17^ and will be assessed by counting the number of patients presenting with one or more complications. The total numbers of complications and each grade will be specified.

A serious complication is defined as a complication which:

1. results in death within 90 days of surgery irrespective of its association with surgery
2. is life-threatening
3. requires unplanned in-patient hospitalization or prolongation of existing hospitalization due to early postoperative complications
4. requires reoperation due to early postoperative complications
5. results in persistent or significant disability or incapacity
6. is otherwise considered medically significant by the investigator

**6.7.2 Expected complications**

**Operative**

- Damage to organ/structure e.g.
- Heart
- Pericardium
- Diaphragm
- Major vessels
- Nerves
- Hemorrhage
- Failure of surgical equipment (robotic system or thoracoscopic equipment, including hardware/software malfunction)

**Postoperative**

- Subcutaneous emphysema
- Pulmonary air leak
- Pneumonia
- Hemorrhage
- Wound infection
- Pleural effusion
- Pneumothorax
- Hypoxemia
- Acute renal failure
- Cerebrovascular attack/stroke
- Disseminated intravascular coagulation
- Sepsis
- Delerium
- Urinary retention

**Other**

- Acute renal failure
- Cardiac arrhythmia
- Cardiac failure
- Ischemic heart disease/myocardial infarction
- Pulmonary embolus
- Respiratory failure
- Urinary tract infection

**6.7.3 Reporting of complications**

Information on all complications will be collected for this trial whether volunteered by the patient, discovered by investigator questioning or detected through physical examination, laboratory test or other investigation.

**6.7.4 Responsibilities for safety reporting**

Complications should be reported based on relevant regulations. The physician in charge should hold accountability and responsibility for the emergency treatment of patients with any degree of complications to ensure patient safety.

**Reporting of serious complications**

Serious complication is defined in section 6.7.1. Serious complication should be recorded and reported in following procedures:

1. The physician in charge or the study monitor who has confirmed any serious complication encountered must report it immediately to the principal investigator (Hecheng Li). If the principal investigator cannot be contacted, the physician in charge or the study monitor must act on his behalf.
2. The principal investigator or his designee must complete the “Serious Complication Report” form and submit it to the IRB for further assessment within 24 hours of becoming aware of the serious complication (Appendix 5).
3. The IRB, along with the principal investigator and other members of the study team, on determining the urgency, importance, causality, and degree of impact of the report, will discuss the necessity of suspending further enrollment or even termination of the study. The discussion will be held within 24 hours of the submission of the “Serious Complication Report” form.

**Reporting of nonserious complications**

For nonserious complications, regular reporting obligations should be followed:

1. The physician in charge or the study monitor who has confirmed any nonserious complication should record the type and degree of the complication in the CRF.
2. The principal investigator, along with other members of the surgery team, will have a review and discussion about the presenting nonserious complications on a weekly basis, to confirm whether there is an under-reporting and misjudgment of the nonserious complications.

# 7. STATISTICAL CONSIDERATIONS

## 7.1 Statistical hypotheses

This one-arm trial aims to test the safety and feasibility of conversion surgery after induction therapy for locally advanced and advanced non-small cell lung cancer. Our hypothesis is that more than 90% patients will reach R0 resection with acceptable surgical-related morbidity, MPR/pCR rate, and 1-year DFS/OS. There may be difference towards DFS/OS between the MPR and non-MPR cohort.

## 7.2 Sample size estimation

Regarding the exploratory nature of this study, a sample size of 30 patients was determined.

## 7.3 Statistical analysis principles

All statistical analyses will be performed using SPSS 22.0 (IBM), R (version3.5.3, R Foundation for Statistical Computing, Vienna, Austria) or GraphPad Prism version 8.0.0 for Windows (GraphPad Software). The significance level for testing differences between the two groups will be set at α=0.05 (bilateral). A significance level of P < 0.05 will be considered statistically significant, and a 95% confidence interval (CI) will be used.

Categorical variables will be summarized by descriptive statistics, including the number of participants, number of events, and percentage. We will use Pearson Chi-squared test or Fisher exact test to compare the difference between the two groups.

Continuous variables following a normal distribution will be presented as mean ± standard deviation (SD), and the Student’s t-test was used for comparison. In cases of noncompliance with the normal distribution, continuous variables will be presented as medians (interquartile range [IQR]) and compared using the Wilcoxon rank-sum test between the two groups.

Regarding the survival data, the Kaplan-Meier approach will be used to calculate OS and DFS at 1, 2, 3, 4, and 5 years. The estimates of median survival time will be provided. Survival curves will be compared using the log-rank test. The Cox proportional hazard regression model will be used to calculate the hazard ratio (HR) and the corresponding 95% CI.

## 7.4 Missing data strategy

Missing data will not be imputed.

## 7.5 Statistical analysis set

An intention-to-treat analysis will be performed. All included participants who meet the eligibility will be included in the final analysis. Patients who do not comply with the protocol will be analyzed in their initial treatment arm. These patients will have to be regularly followed up according to the protocol.

## 7.6 Interim analysis

No scheduled interim analysis will be conducted in this study.

# 8. ETHICAL CONSIDERATIONS

This study will be carried out upon approval of the IRB and in compliance with the Helsinki Declaration, applicable GCP guidelines, and applicable laws and regulations. Patients can participate in the study only after having signed the informed consent form in full awareness of the objectives of the study and the risks involved. Informed written consent will be obtained from the patients prior to randomization into the trial. The right of a patient to refuse participation without giving reasons must be respected. The patient must remain free to withdraw at any time from the trial without giving reasons and without prejudicing his/her further treatment. All information collected during the course of the trial will be kept strictly confidential.

## 8.1 Responsibilities of investigators

The investigators are responsible for the conduction of this study. The investigators will ensure the implementation of this study in accordance with the study protocol and in compliance with the Declaration of Helsinki, as well as domestic ethical guiding principles and applicable regulatory requirements. It is specially noted that, the investigators must ensure that only subjects who provide informed consent and fulfill the eligibility criteria can be enrolled in this study.

## 8.2 Compensation

Health injury incurred by participating in the present clinical trial will be treated appropriately according to the symptoms in the way as normal medical care and will be covered by the national health insurance scheme. Any medical expenses not covered by the national health insurance scheme will be incurred by the patient. As this is a clinician-led trial there are no arrangements for no-fault compensation.

# 9. REFERENCES

1. Sung H, Ferlay J, Siegel RL, et al. Global Cancer Statistics 2020: GLOBOCAN Estimates of Incidence and Mortality Worldwide for 36 Cancers in 185 Countries. *CA Cancer J Clin.* 2021;71(3):209-249.

2. Mao Y, Yang D, He J, Krasna MJ. Epidemiology of Lung Cancer. *Surg Oncol Clin N Am.* 2016;25(3):439-445.

3. Krupnick AS. Neoadjuvant Multimodality Induction Therapy for Locally Advanced Initially Unresectable Lung Cancer: A New Hope. *Ann Surg.* 2022;275(3):e603-e604.

4. Lei J, Zhao J, Gong L, et al. Neoadjuvant Camrelizumab Plus Platinum-Based Chemotherapy vs Chemotherapy Alone for Chinese Patients With Resectable Stage IIIA or IIIB (T3N2) Non-Small Cell Lung Cancer: The TD-FOREKNOW Randomized Clinical Trial. *JAMA Oncol.* 2023;9(10):1348-1355.

5. Sun C, Wang X, Xu Y, et al. Efficiency and safety of neoadjuvant PD-1 inhibitor (sintilimab) combined with chemotherapy in potentially resectable stage IIIA/IIIB non-small cell lung cancer: Neo-Pre-IC, a single-arm phase 2 trial. *EClinicalMedicine.* 2024;68:102422.

6. Provencio M, Nadal E, Gonzalez-Larriba JL, et al. Perioperative Nivolumab and Chemotherapy in Stage III Non-Small-Cell Lung Cancer. *N Engl J Med.* 2023;389(6):504-513.

7. Zheng J, Li Y, Jin C, et al. Efficacy and surgical safety of sequential surgical resection after pembrolizumab plus chemotherapy for initial unresectable stage IIIB non-small cell lung cancer. *Lung Cancer.* 2023;184:107326.

8. Deng H, Liu J, Cai X, et al. Radical Minimally Invasive Surgery After Immuno-chemotherapy in Initially-unresectable Stage IIIB Non-small cell Lung Cancer. *Ann Surg.* 2022;275(3):e600-e602.

9. Shepherd FA, Ginsberg R, Patterson GA, et al. Is there ever a role for salvage operations in limited small-cell lung cancer? *J Thorac Cardiovasc Surg.* 1991;101(2):196-200.

10. Sonobe M, Yutaka Y, Nakajima D, et al. Salvage Surgery After Chemotherapy or Chemoradiotherapy for Initially Unresectable Lung Carcinoma. *Ann Thorac Surg.* 2019;108(6):1664-1670.

11. National Comprehensive Cancer Work Clinical Practice Guidelines in Oncology (NCCN Guidelines) for Non-Small Cell Lung Cancer Version.3 2019. 2019. <https://www.nccn.org/home>.

12. Goldstraw P, Chansky K, Crowley J, et al. The IASLC Lung Cancer Staging Project: Proposals for Revision of the TNM Stage Groupings in the Forthcoming (Eighth) Edition of the TNM Classification for Lung Cancer. *Journal of thoracic oncology : official publication of the International Association for the Study of Lung Cancer.* 2016;11(1):39-51.

13. Travis WD, Brambilla E, Burke AP, Marx A, Nicholson AG. *WHO Classification of Tumours of the Lung, Pleura, Thymus, and Heart.* Lyon: International Agency for Research on Cancer; 2015.

14. Aaronson NK, Ahmedzai S, Bergman B, et al. The European Organization for Research and Treatment of Cancer QLQ-C30: a quality-of-life instrument for use in international clinical trials in oncology. *Journal of the National Cancer Institute.* 1993;85(5):365-376.

15. Bergman B, Aaronson NK, Ahmedzai S, Kaasa S, Sullivan M. The EORTC QLQ-LC13: a modular supplement to the EORTC Core Quality of Life Questionnaire (QLQ-C30) for use in lung cancer clinical trials. EORTC Study Group on Quality of Life. *European journal of cancer (Oxford, England : 1990).* 1994;30a(5):635-642.

16. EuroQol--a new facility for the measurement of health-related quality of life. *Health policy (Amsterdam, Netherlands).* 1990;16(3):199-208.

17. Dindo D, Demartines N, Clavien PA. Classification of surgical complications: a new proposal with evaluation in a cohort of 6336 patients and results of a survey. *Annals of surgery.* 2004;240(2):205-213.

# 10. APPENDICES

Appendix 1 The eighth edition of the TNM classification for lung cancer

Appendix 2 The American Society of Anesthesiologists (ASA) Physical Status Classification

Appendix 3 English model of informed consent form

Appendix 4 The Clavien-Dindo classification of surgical complication

Appendix 5 English model of serious complication report form

## Appendix 1 The eighth edition of the TNM classification for lung cancer

(From Goldstraw P, et al. J Thorac Oncol. 2016 Jan;11(1):39-51.)

| **T: Primary tumor** |  |
| --- | --- |
| Tx | Primary tumor cannot be assessed or tumor proven by presence of malignant cells in sputum or bronchial washings but not visualized by imaging or bronchoscopy |
| T0 | No evidence of primary tumor |
| Tis | Carcinoma in situ |
| T1 | Tumor ≤3 cm in greatest dimension surrounded by lung or visceral pleura without bronchoscopic evidence of invasion more proximal than the lobar bronchus (i.e., not in the main bronchus) |
| T1a (mi) | Minimally invasive adenocarcinoma |
| T1a | Tumor ≤1 cm in greatest dimension |
| T1b | Tumor >1 cm but ≤2 cm in greatest dimension |
| T1c | Tumor >2 cm but ≤3 cm in greatest dimension |
| T2 | Tumor >3 cm but ≤5 cm or tumor with any of the following features:  - Involves main bronchus regardless of distance from the carina but without involvement of the carina  - Invades visceral pleura  - Associated with atelectasis or obstructive pneumonitis that extends to the hilar region, involving part or all of the lung |
| T2a | Tumor >3 cm but ≤4 cm in greatest dimension |
| T2b | Tumor >4 cm but ≤5 cm in greatest dimension |
| T3 | Tumor >5 cm but ≤7 cm in greatest dimension or associated with separate tumor nodule(s) in the same lobe as the primary tumor or directly invades any of the following structures:  chest wall (including the parietal pleura and superior sulcus tumors), phrenic nerve, parietal pericardium |
| T4 | Tumor >7 cm in greatest dimension or associated with separate tumor nodule(s) in a different ipsilateral lobe than that of the primary tumor or invades any of the following structures:  diaphragm, mediastinum, heart, great vessels, trachea, recurrent laryngeal nerve, esophagus, vertebral body, and carina |
| **N: Regional lymph node involvement** |  |
| Nx | Regional lymph nodes cannot be assessed |
| N0 | No regional lymph node metastasis |
| N1 | Metastasis in ipsilateral peribronchial and/or ipsilateral hilar lymph nodes and intrapulmonary nodes, including involvement by direct extension |
| N2 | Metastasis in ipsilateral mediastinal and/or subcarinal lymph node(s) |
| N3 | Metastasis in contralateral mediastinal, contralateral hilar, ipsilateral or contralateral scalene, or supraclavicular lymph node(s) |
| **M: Distant metastasis** |  |
| M0 | No distant metastasis |
| M1 | Distant metastasis present |
| M1a | Separate tumor nodule(s) in a contralateral lobe; tumor with pleural or pericardial nodule(s) or malignant pleural or pericardial effusion |
| M1b | Single extrathoracic metastasis |
| M1c | Multiple extrathoracic metastases in one or more organs |

| **T/M** | Subgroup | **N0** | **N1** | **N2** | **N3** |
| --- | --- | --- | --- | --- | --- |
| **T1** | **T1a**≤1cm | IA1 | IIB | IIIA | IIIB |
|  | 1cm<**T1b**≤2cm | IA2 | IIB | IIIA | IIIB |
|  | 2cm<**T1c**≤3cm | IA3 | IIB | IIIA | IIIB |
| **T2** | 3cm<**T2a**≤4cm | IB | IIB | IIIA | IIIB |
|  | 4cm<**T2b**≤5cm | IIA | IIB | IIIA | IIIB |
| **T3** | 5cm<**T3**≤7cm | IIB | IIIA | IIIB | IIIC |
| **T4** | 7cm<**T4** | IIIA | IIIA | IIIB | IIIC |
| **M1** | **M1a** | IVA | IVA | IVA | IVA |
|  | **M1b** | IVA | IVA | IVA | IVA |
|  | **M1c** | IVB | IVB | IVB | IVB |

## Appendix 2 The American Society of Anesthesiologists (ASA) Physical Status Classification

According to the patients' physical status and surgical risk before anesthesia, the American Society of Anesthesiologists (ASA) has categorized patients into 5 levels (I-V levels) as follows:

Class I: Well-developed patients with physical health and normal function of various organs, having a perioperative mortality rate of 0.06%-0.08%.

Class II: Patients with mild complications and good functional compensation in addition to surgical diseases, having a perioperative mortality rate of 0.27%-0.40%.

Class III: Patients with severe complications, restricted physical activity, but still capable of coping with day-to-day activities, having a perioperative mortality rate of 1.82%-4.30%.

Class IV: Patients with serious complications, who have lost ability of day to day activity, often with life threatening conditions, having a perioperative mortality rate of 7.80%-23.0%.

Class V: Moribund patients receiving a surgery or not, little chance for survival, having a perioperative mortality rate of 9.40%-50.70%.

Generally, Class I/II patients are considered good for anesthesia and surgical tolerance, with a smooth anesthesia process. Class III patients are exposed to some anesthesia risks, and therefore good preparations should be fully made before anesthesia, and effective measures should be taken to prevent potential complications during the anesthesia. Class IV patients are exposed to the most risks, even if good preoperative preparations are made, with perioperative mortality rate is being very high. Class V patients are moribund patients and should not undergo an elective surgery.

## Appendix 3 English model of informed consent form

(The original informed consent form is written in Chinese. It is translated to Chinese here in the appendices.)

**The Study of Safety and Feasibility of Surgery After Conversion Therapy for**

**Locally Advanced and Advanced NSCLC**

**Patient informed consent form**

**Introduction**

We sincerely invite you to participate in the study entitled " Safety and Feasibility of Surgery After Conversion Therapy for Locally Advanced and Advanced NSCLC ". The objective of this study is to evaluate the safety and feasibility of surgery after conversion therapy for locally advanced and advanced non-small cell lung cancer. Before you decide to participate, it is essential to understand the purpose and content of the study. Please read this informed consent carefully and discuss it with your doctor, family and friends. If there is anything unclear, or you would like to know more, please ask your doctor or contact the person listed at the end of this informed consent directly.

**What is conversion therapy?**

Conversion therapy converted NSCLC from initially unresectable to potentially resectable status because of a favorable response to induction therapy. Induction therapy include chemotherapy, chemotherapy plus immunotherapy, and targeted therapy.

**What is the purpose of the study?**

Lung cancer is the most common malignant tumor in our country. Approximately 80% of patients with NSCLC present with either metastatic or locally advanced disease that is not initially amenable to resection. Thanks to the advances in drug therapy and radiotherapy for NSCLC, conversion surgery after induction therapy for initially unresectable NSCLC was proposed and evaluated rapidly. The induction therapy is potential to convert NSCLC from initially unresectable to potentially resectable status because of a favorable response. However, it requires strict operation indications and patient screening. Furthermore, this concept of treatment still needs evidence from prospective studies to prove its safety and feasibility.

We carry out this prospective clinical trial to evaluated the safety and feasibility of surgery after conversion therapy for initially unresectable locally advanced and advanced NSCLC patients.

**How many people will take part in the study?**

About 30 people will take part in this study.

**What will happen if I take part in this research study?**

If you agree to participate, you will receive convention surgery for NSCLC. Surgery will be performed under general anesthesia with double-lumen endotracheal intubation. Robot-assisted thoracic surgery (RATS), video-assisted thoracic surgery (VATS), and thoracotomy are optional choice of surgical approach. The specifics of each operation will be at the discretion of the operating surgeon (e.g., port placement, lymph node dissection), as will the decision to convert to an open operation.

**Do I have to participate in the study?**

No. Participation in the study is voluntary, not forced. After you participate in this trial, you will remain free to withdraw from the trial at any time without giving reasons and without prejudicing any further treatment. If you decide to participate, you will be asked to sign an informed consent form. You will retain a copy of this consent form and this introduction.

**What side effects or risks can I expect from being in the study?**

You may have side effects while on the study. The main risks will be associated with surgery and its related complications. Everyone taking part in the study will be watched carefully for any side effects. However, doctors don’t know all the side effects that may happen. Side effects may be mild or very serious. Your health care team may give you medicines to help lessen side effects. The side effects of video-assisted lobectomy and robotic-assisted lobectomy are generally similar according to our previous clinical experience and published retrospective studies.

You should talk to your study doctor about any side effects that you have while taking part in the study.

The risks of surgery include:

**Likely**

- Air leaking from the part of the lung that was operated on
- Cough and phlegm
- Partial collapse of lung

**Less likely**

- Pneumonia
- Pleural effusion (fluid in the chest cavity, usually temporary)
- Heart attack, heart failure, or irregular heartbeat

**Rare**

- Difficulties breathing that may require a ventilator (breathing machine) for days or weeks after surgery
- Infection in the area around the lung, wound infection or blood infection
- Bleeding
- Poor healing of the skin and/or muscles in the chest
- Blood clots in the legs and/or lung
- Cerebrovascular attack/stroke
- Urinary tract infection

For more information about the side effects, you can ask your doctor.

**Will my medical information be kept private?**

We will do our best to make sure that the personal information in your medical record will be kept private. However, we cannot guarantee total privacy. Your personal information may be given out if required by law.

Any information transmitted electronically will be renamed to ensure confidentiality. Information on all computers will be protected with a password.

If information from this study is published or presented at scientific journals or meetings, your name and other personal information will not be used.

**What are the costs of taking part in this study?**

You and/or your health plan/ insurance company will need to pay for some or all of the costs of treating your cancer in this study. Some health plans will not pay these costs for people taking part in studies. Check with your health plan or insurance company to find out what they will pay for. Taking part in this study may or may not cost your insurance company more than the cost of getting regular cancer treatment. If you are randomized to the robotic-assisted lobectomy group, generally you will spend more for your treatment as robotic-assisted lobectomy is currently not reimbursed in the national health insurance system. You will not be paid for taking part in this study.

**Who is conducting this study?**

This study is conducted by the department of thoracic surgery, Ruijin Hospital affiliated to Shanghai Jiao Tong University School of Medicine.

**Who should I contact for more information?**

After reading this introduction and discussing it with your doctor, family and friends, if you have further questions or concerns, please contact following researchers for help:

Researcher: Runsen Jin, Chengqiang Li

Phone number: 18516266098, 13524282905, 64370045-362388

Address: Department of Thoracic Surgery, 7th Floor, Building 6, Ruijin Hospital, No.197 Ruijin Er Road, Huangpu District, Shanghai

**Has this study been approved?**

Yes. This study has been approved by the ethics committee of Ruijin Hospital, Shanghai Jiao Tong University School of Medicine (approval number, 2020.344).

Anyone with questions about this study can contact the following people directly:

Researcher: Runsen Jin, Chengqiang Li

Phone number: 18516266098, 13524282905, 64370045-362388

I have read the above content carefully and agree to participate in the study.

Patient’s name: _______________

Patient’s signature: _______________ Date: _______________

Surgeon’s name: _______________

Surgeon’s signature: _______________ Date: _______________

## Appendix 4 The Clavien-Dindo classification of surgical complication

(From Dindo D, et al. Ann Surg. 2004 Aug;240(2):205-13.)

| Classification of surgical complications | |
| --- | --- |
| **Grade** | **Definition** |
| Grade I | Any deviation from the normal postoperative course without the need for pharmacological treatment or surgical, endoscopic, and radiological interventions  Allowed therapeutic regimens are: drugs as antiemetics, antipyretics, analgesics, diuretics, electrolytes, and physiotherapy. This grade also includes wound infections opened at the bedside |
| Grade II | Requiring pharmacological treatment with drugs other than such allowed for grade I complications  Blood transfusions and total parenteral nutrition are also included |
| Grade III | Requiring surgical, endoscopic or radiological intervention |
| Grade IIIa | Intervention not under general anesthesia |
| Grade IIIb | Intervention under general anesthesia |
| Grade IV | Life-threatening complication (including CNS complications)* requiring IC/ICU management |
| Grade IVa | Single organ dysfunction (including dialysis) |
| Grade IVb | Multiorgan dysfunction |
| Grade V | Death of a patient |
| Suffix “d” | If the patient suffers from a complication at the time of discharge, the suffix “d” (for “disability”) is added to the respective grade of complication. This label indicates the need for a follow-up to fully evaluate the complication. |
| *Brain hemorrhage, ischemic stroke, subarrachnoidal bleeding, but excluding transient ischemic attacks.  CNS, central nervous system; IC, intermediate care; ICU, intensive care unit. | |

## Appendix 5 English model of serious complication report form

(The original serious complication report form is written in Chinese. It is translated to Chinese here in the appendices.)
