# Supplementary material for: Conversion Surgery After Induction Therapy for Initially Unresectable Stage III Non-small Cell Lung Cancer: A Proof-of-Concept Trial
Source: Ann Surg Oncol. 2026 Mar 25;33(7):6242–51. doi: 10.1245/s10434-026-19461-z (PMC13242421; doi:10.1245/s10434-026-19461-z)
Supplement: Supplementary file 2 — Supplementary file2 (DOCX 28 KB) [file 10434_2026_19461_MOESM2_ESM.docx]

**RNA sequencing analysis**

A total of 11 tumor samples were collected for bulk-RNA sequencing analysis (Supplemental Table 1). RNA was extracted from samples with the Biozol RNA Miniprep Kit (BW-R7311, Peiwo) according to the manufacturer’s instructions. RNA quantity was analyzed using Qubit 4.0 (Invitrogen) and quality examined by electrophoresis on a denaturing agarose gel. Total RNA was first depleted of rRNA using the Ribo-off rRNA Depletion Kit (Human/Mouse/Rat) (N406-02, Vazyme). Sequencing libraries were generated using the VVAHTS® Universal V8 RNA-seq Library Prep Kit for Illumina (NR605-0, Vazyme). The libraries were sequenced as 151-bp paired-end reads using Illumina NovaSeq 6000 platform. Enrichment of mRNA, library construction, sequencing and data analysis were performed by Shanghai Xu Ran Biotechnology Co., Ltd (http://www.xurangene.com).

For raw reads, Illumina adapter sequences were trimmed using Skewer (version 0.2.2). And the quality checking was performed using FastQC (version 0.11.5). The clean reads were aligned to the GRCh38 reference genome GRCh38 using STAR (version 2.5.3a). StringTie (version 1.3.1c) was used to assemble transcripts and generate gene expression data. Differentially expression analysis was performed by the R package DESeq2 (version 1.16.1). Differentially expressed genes exhibiting two-fold changes and P values ≦ 0.05 were selected.

For gene set enrichment analysis (GSEA), results for all protein-coding genes were ranked by fold change evaluated with the ‘GSEA’ algorithm.^1^ ‘Hallmark’ and ‘KEGG’ gene sets were acquired from MSigDb. We filtered the GSEA results based on the criterion of P value ≦0.05. We visualized candidate pathways based on the normalized enrichment score from the filtered list.

For gene set variation analysis (GSVA), the top ten differentially expressed genes (DEGs) in the top five enriched pathways by GSEA composed the gene set for ‘GSVA’ algorithm.^2^ The GSVA score of MPR and non-MPR samples of ICT group was presented as mean ± standard deviation (SD), and the student’s t-test was used for comparison. The result was visualized by histogram.

**Reference**

1. Subramanian A, Tamayo P, Mootha VK, et al. Gene set enrichment analysis: a knowledge-based approach for interpreting genome-wide expression profiles. Proc Natl Acad Sci U S A. Oct 25 2005;102(43):15545-50.

2. Hanzelmann S, Castelo R, Guinney J. GSVA: gene set variation analysis for microarray and RNA-seq data. BMC Bioinformatics. Jan 16 2013;14:7.

**Supplemental Table 1**

| **Patient ID** | **Sample ID** | **Group** | **Subgroup** | **Histology** | **cTNM stage** | **ypTNM stage** |
| --- | --- | --- | --- | --- | --- | --- |
| 02 | BS-02 | ICT | pCR | SCC | cT4N2M0 | ypT0N0M0 |
| 02 | PT-02 | ICT | pCR | SCC | cT4N2M0 | ypT0N0M0 |
| 03 | PT-03 | ICT | Non-MPR | SCC | cT4N3M0 | ypT2N1M0 |
| 10 | BS-10 | ICT | pCR | SCC | cT4N2M0 | ypT0N0M0 |
| 10 | PT-10 | ICT | pCR | SCC | cT4N2M0 | ypT0N0M0 |
| 16 | PT-16 | ICT | Non-MPR | SCC | cT4N2M0 | ypT2aN0M0 |
| 20 | PT-20 | ICT | Non-MPR | SCC | cT4N2M0 | ypT2bN0M0 |
| 21 | BS-21 | ICT | pCR | SCC | cT3N2M0 | ypT0N0M0 |
| 21 | PT-21 | ICT | pCR | SCC | cT3N2M0 | ypT0N0M0 |
| 28 | PT-28 | ICT | MPR, non-pCR | SCC | cT4N2M0 | ypT4N0M0 |
| 29 | PT-29 | ICT | MPR, non-pCR | SCC | cT2bN2M0 | ypT1cN0M0 |

Abbreviations: SCC, squamous cell carcinoma.
